# Current Status of Clinical Engineer Anesthesia Assistants and Their Effect on Labor Task Shifting in Japan: A Prospective Observational Study in a Single Institute

**DOI:** 10.31662/jmaj.2020-0100

**Published:** 2021-03-26

**Authors:** Yusuke Naito, Hideaki Kawanishi, Michinori Kayashima, Sawako Okamoto, Tomoaki Imamura, Hitoshi Furuya, Junji Egawa, Masahiko Kawaguchi

**Affiliations:** 1 Department of Anesthesiology, Nara Medical University, Kashihara, Japan; 2 Department of Medical Technology, Nara Medical University, Kashihara, Japan; 3 Department of Public Health, Nara Medical University, Kashihara, Japan

**Keywords:** Anesthesia, Task Shifting, Clinical Engineer Anesthesia Assistants

## Abstract

**Introduction:**

Anesthesiologists are in short supply across the world, resulting in perpetually long working hours. To reduce the burden on anesthesiologists, tasks that can be performed by non-physicians must be shifted to other medical staff. In hospitals, clinical engineers can work as anesthesia assistants and perform some of the duties of anesthesiologists. This study aimed to evaluate the effect of task shift performed by clinical engineer anesthesia assistants (CEAAs).

**Methods:**

This was a 1-month prospective observational study that included 33 anesthesiologists (11 fellows and 22 certified anesthesiologists) and 11 CEAAs. The total activity and anesthesia times were extracted from the attendance record as indices of the anesthesiologists' work status. The CEAAs recorded the duration of work performed on behalf of the anesthesiologists as task shift time. The task shift rate was evaluated as follows: task shift time/(task shift time + total activity time) and task shift time/(task shift time) + (total anesthesia time).

**Results:**

The study period consisted of 19 weekdays. The average daily activity time of the anesthesiologists was 10.1 h, and the average anesthesia time was 8.5 h. The CEAAs performed a total of 546.8 h of task shift. The defined task shift rate was 20.1% when the total activity time was the denominator and 23.1% when the anesthesia time was the denominator.

**Conclusions:**

CEAAs might be effective in reducing the working hours of anesthesiologists through task shift. Their taking over a portion of the anesthesiologists' duties may allow the anesthesiologists to work more efficiently.

## Introduction

Various countries worldwide are experiencing a lack of anesthesiologists ^(1), (2)^. This is especially true for developing countries, where only 0.1 anesthesiologists per 100,000 people are available ^(3)^. Anesthesiologists play an important role in increasing the safety of the perioperative period. Previous studies have identified the relationship between perioperative mortality and the number of anesthesiologists as significant. As a way around limited resources, occupations to assist anesthesiologists, such as certified registered nurse anesthetists (CRNAs) in the United States, have been created ^(4)^. In the United States, nearly half of the anesthesia care providers are CRNAs. CRNAs play an important role in conducting medical procedures, such as tracheal intubation of patients and administering anesthetic drugs. However, in several countries, including Japan, these medical procedures are limited only to physicians; only under exceptional circumstances can these be handled by the medical staff. To address this problem, solutions that can be applied across cultures and legal systems are needed.

To keep up with the increasing number of surgeries while maintaining the current medical standards, reallocating anesthesiologists' tasks to other medical staff, called task shifting in Japan, is gaining popularity. The Ministry of Health, Labour and Welfare has developed a plan to improve the balance of supply and demand of physicians based on the assumption that 7% of the tasks will be shifted in the future. However, to date, no study has exclusively evaluated the task shift rate. Task shifting is mainly applied in the nursing profession and has been increasing in popularity ^(5)^. However, our hospital has been educating and working with clinical engineers as anesthesia assistants (CEAAs) for > 10 years.

Unlike nurses, CEAAs cannot perform invasive clinical medical practices (e.g., inserting intravenous or arterial lines, intubating the trachea of the patient, or administering drugs), but they demonstrate high specialty in the operation and maintenance of biomedical equipment. They can support anesthesiologists in different aspects compared with nurses. Several studies have evaluated the effect of task shifting on nurses ^(5)^, but research on the efficacy of CEAAs is lacking. This study aimed to evaluate the efficacy of CEAAs by calculating task-shifted time.

## Materials and Methods

### Participants

This prospective observational study was approved by the Ethics Committee of Nara Medical University (approval number: 2252). The study included 33 anesthesiologists (11 fellows and 22 certified anesthesiologists) and 11 CEAAs. Residents were not involved in this study. After consultation with the ethics committee, written informed consent was waived considering the nature of the study. Alternatively, after explaining the research protocol, verbal consent was obtained during staff meeting. For those who did not attend the meeting, verbal informed consent was obtained individually after the meeting. The observation was conducted in our hospital, which has 14 operating rooms and 1 hybrid room, and the anesthesiologists are involved in approximately 6000 surgeries per annum.

### Procedure

**Figure 1.**
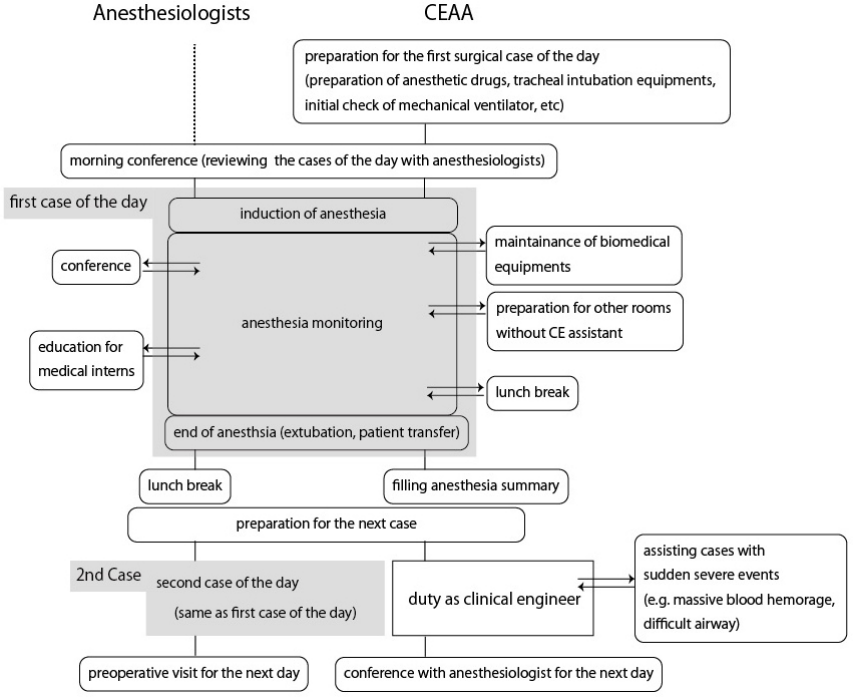
Typical daily labor flowchart for anesthesiologists and CEAAs.

A 1-month observational study was conducted from September 1, 2019, to September 30, 2019. Anesthesiologists' working records were collected using electronic timecard records each day during the study period. We excluded the records of anesthesiologists who were not in the hospital, that is, if the anesthesiologists were working for the outpatient clinic, ICU, palliative care medicine unit, or pain clinic center. Each day, the CEAAs completed a daily report for this study, describing their work, and the actual dedicated time. Because CEAAs not only worked as anesthesia assistants but also performed routine work as clinical engineers, such as preparing continuous hemodiafiltration circuits in the ICU, we predefined task shift contents as shown in Table 1. Figure 1 shows the typical workflow experienced by anesthesiologists and CEAAs.

**Table 1.** Daily Activity of CEAAs and Definition of Task Shift Contents.

Category	Descriptions	Included as task shift work
Duty as a clinical engineer	Equipment maintenance and management unrelated to anesthesia care (e.g., syringe pump maintenance in the general ward), conference between CEs, CHDF priming, PMX circuit assembly, ventilator maintenance, blood gas analysis assistance, respiratory settings in the ICU	No
Conferences	Pre-anesthesia conferences, joint conferences with other departments, on behalf of anesthesiologists	Yes
Equipment maintenance	Equipment maintenance and management related to anesthesia practice (e.g., transesophageal echocardiography and fiberoptic bronchoscopy)	Yes
Anesthesia preparation	Preparation of airway devices (e.g., endotracheal tubes, laryngoscope, or video laryngoscopes), assembly of pressure lines, preparation of CV catheters, pre-use anesthesia machine checking, setting of monitors, preparation of anesthetic drugs	Yes
Documents	Filling case summary for submission to JSA, storage of intraoperative images (TEE, etc.)	Yes
Anesthesia assistance	Anesthesia management in the presence of attending anesthesiologists (e.g., completing the electronic anesthetic record, preparing a drug, and adjusting sonography settings) troubleshooting of biomedical equipment during anesthesia	Yes
Sole anesthesia monitoring	Sole monitoring in the absence of an anesthesiologist when the vital signs are stable during surgery	Yes
Others	Handing out arterial blood sampling and reporting the results, teaching residents and students	Yes

### Data analysis

We defined activity and anesthesia times as indices of the working status of anesthesiologists. The activity time was defined as the total labor performed in the hospital, which was calculated by subtracting the 45-min break time from the length of stay in the hospital. The anesthesia times were calculated on the basis of the time during which each anesthesiologist was providing anesthesia management. This was based on electronic anesthesia records. For anesthesiologists consecutively working during the night shift after a day shift, we excluded labor during the night shifts and included the overtime hours if the time exceeded 8:30 a.m. the next morning. Labor during weekends and holidays was also excluded from the study even if they were working during these hours. The task shift time of CEAAs was classified into three categories: morning off-duty hours (until 8:30 a.m.), day shift hours (from 8:30 a.m. to 5:15 p.m.), and evening off-duty hours (after 5:15 p.m.).

When the CEAAs performed task shift activities, the time spent was recorded. If the work was fully performed by the CEAAs, the total time dedicated to the work was counted as the task shift time. Alternatively, when the work was performed in collaboration with the attending anesthesiologist, half of the consumed time was counted as task shift time. Finally, the task shift rate was calculated as the ratio of the total task shift time to the total number of anesthesiologists' working hours. Because we defined two indices (active and anesthesia times) for the working status of anesthesiologists, the formulas of the task shift rates were as follows: task shift rate 1 = total task shift time/(total task shift time) + (total active time); task shift rate 2 = total task shift time/(total task shift time) + (total anesthesia time).

## Results

The observation period consisted of 19 weekdays. During this time, 228 records were extracted from the electronic timecard system after excluding work other than anesthesia management (ICU work; university work, such as meetings, outside work, and academic activities). Table 2 shows the anesthesiologists' activity and anesthesia times for the observation period. The anesthesiologists reported an average activity time of 10.1 h and an average anesthesia time of 8.5 h per day. The anesthesiologists' overtime work of 2.1 h comprised an average of 0.82 h of morning off-duty work and 1.28 h of evening off-duty work.

**Table 2.** Results of Anesthesiologists' Working Hours.

		Average	Total over 19 days
Day shift duties			
Number of anesthesiologists (/day)		10.5 ± 1.7	
	Active time	10.1 ± 1.9	2005.8
	Anesthesia time	8.5 ± 1.1	1704.0
Overtime duty after night shift			
Number of anesthesiologists (/day)		1.5 ± 1.0	
	Active time	5.8 ± 2.5	163.9
	Anesthesia time	3.9 ± 2.1	111.2
Total			
	Active time	-	2169.7
	Anesthesia time	-	1815.2

Active time: the actual time that anesthesiologists were in the hospital. Anesthesia time: time working purely for anesthetic management

An average of 7.2 CEAAs worked each day during the observation period. Table 3 shows the individual task details and the actual task shift time performed by the CEAAs. The total task shift time was 546.8 h, and the average task shift time was 4.0 h per CEAA per day. The average task shift time categorized by working hours was as follows: 0.65, 3.3, and 0.03 h for morning off-duty work, on-shift hours, and evening off-duty work, respectively. According to the predefined clinical engineer task shift rate, task shift rate 1 (based on activity time) was 20.1% (=546.8/(546.8 + 2169.7)), and task shift rate 2 (based on anesthesia time) was 23.1%.

**Table 3.** Detailed Task-Shifted Time by CEAAs.

	Number of records	Actual hours	Percentage	Task shift time (h)
Duties as a clinical engineer	121	178.9	13.5	0
Conferences	123	50.9	3.9	3.8
Equipment maintenance	31	54.0	4.1	39.2
Anesthesia preparation	331	146.6	11.1	87.3
Documents	30	11.6	0.9	6.3
Anesthesia assistance	270	716.4	54.2	261.9
Sole anesthesia monitoring	322	145.7	11.0	145.7
Others	21	16.6	1.3	2.6
Total time		1320.7		546.8		

The numbers are shown as the average number of hours, categorized by the work predefined in Table 1. All task times were summed after multiplying by 0 if the work was mainly performed by anesthesiologists, 0.5 for the work performed in cooperation with anesthesiologists, or 1.0 if the work was performed solely by the CEAAs.

## Discussion

To the best of our knowledge, this study is the first to objectively evaluate the task shift rate. The task shift rates of CEAAs in this study were over 20%. The average daily working time for anesthesiologists was 10.1 h, which could be calculated as 50.5 h/week. However, if not for CEAAs, the workload for anesthesiologists per week would be 61.3 h, which would exceed the restriction level stipulated by the Labor Standards Act that is in effect until 2024 ^(6)^. Therefore, the task shifting from anesthesiologists to CEAAs might be effective for reducing the workload of anesthesiologists.

Physicians' long working hours can affect performance ^(7)^, increase malpractice ^(8), (9)^, cause clashes with staff ^(10)^, and result in failed procedures ^(11)^ that can affect patient safety. In addition, long-term overtime can cause various health problems, such as coronary events or cerebral strokes. A systematic review evaluating more than 600,000 individuals revealed that those working > 55 h/week are more likely to experience coronary events than those who work < 55 h ^(12)^. Another 7-year cohort study including more than 53,000 individuals revealed a 33% higher risk of cerebral stroke for people working ≥ 55 h than those working for 30-45 h ^(13)^.

Japan is experiencing a lack of anesthesiologists; at present, there are 8,781 board-certified anesthesiologists in the country, corresponding to a rate of 6.9 per 100,000 people. This rate is only approximately 25% of that in the United States, where 52,802 board-certified physician anesthesiologists, 54,000 CRNAs, and 2,000 anesthesia assistants, working together as anesthesia caregivers, combine for a rate of 33.3 anesthesia caregivers per 100,000 people. One reason that Japan is facing a lack of anesthesia caregivers is that performing medical procedures is strictly restricted by Japanese law, and anesthesia assistants are unlikely to grow in number. To keep up with the increasing number of surgeries while maintaining the current medical standards, reallocating anesthesiologists' tasks to other medical staff, or task shifting, is important in Japan.

In this study, task shift work of CEAAs included monitoring vital signs during anesthesia, preparation of anesthetic drugs, maintenance of biomedical equipment, and attending meetings on behalf of anesthesiologists at conferences. The law regarding clinical engineers in Japan defines the duties of a clinical engineer as “the monitoring, operation and maintenance of life support equipment under the assistance of a physician” (Article 2-2). However, the law does not state specific medical actions. Thus, it is unclear to what extent clinical engineers can assist anesthesiologists without violating the current law. Recently, the Ministry of Health, Labour and Welfare has summarized “Tasks that can be performed under the current law” in the “3rd Study Group on Promoting Task Shifting/Sharing to Promote Work Style Innovation among Physicians,” which takes task shifting into consideration ^(14)^. As a result of repeated discussions, the following tasks, which can be performed by clinical engineers without violating the current law, were identified as specific examples of task shift duty: blood collection from inserted arterial catheters, assistance of anesthesia preparation before surgery (preparation of anesthesia equipment, tracheal intubation equipment, and anesthetic drugs), assistance in anesthesia management during surgery (monitoring vital signs and filling in anesthesia records), and assistance in anesthesia management after surgery (organizing various lines and preparation for transfer).

Conversely, it should be noted that the administration of drugs, tracheal intubation, insertion of gastric tubes, and securing of peripheral venous routes are not permitted under the current law even under the direction of a physician. Therefore, when using this system to allocate tasks from physicians, it is necessary for both physicians and clinical engineers to understand the extent to which these tasks can be allocated.

In addition to tasks related to anesthesia, the task shift works in this study included other contents, such as paperwork and maintenance of biomedical equipment. These are tasks originally performed by medical office workers and clinical engineers and thus reallocating them might not be pure task shifting from anesthesiologists to CEAAs. However, in many hospitals where medical clerks and clinical engineers are not available exclusively, these tasks are performed by anesthesiologists. Reflecting on that situation, we included these tasks in this study. We have described in detail the contents of these tasks so that other hospitals can refer to our results and assess their own task shift rates.

The total task shift time for anesthesia management reported in this study was 862.1 h. Of these hours, 716.4 h were spent in cooperation with the anesthesiologist and 145.7 h were for sole monitoring in the absence of the anesthesiologist. We included anesthesia time in cooperation with anesthesiologists because CEAAs and anesthesiologists perform different tasks. For example, in cases of massive bleeding, anesthesiologists can focus on the surgical site, perform blood transfusion, administer drugs, and discuss the situation with surgeons, whereas CEAAs can prepare blood transfusions, prepare drugs, complete electronic anesthetic records, and call for help from other staff. Moreover, when anesthesiologists administer drugs, they verbalize the drug name and dose to clinical engineers. The CEAA verbally repeats the drug name and dose to confirm the procedure and then records this information in the electronic anesthetic record. This works as a way to double-check the name and dose when administering drugs to patients, making the anesthesia management process safe. We believe that this cooperation is more effective from the viewpoint of medical safety than that of task shift. Finally, 146 h of sole anesthetic management were performed by CEAAs when the patient's vital signs were stable. This practice enabled anesthesiologists to briefly leave the room for various reasons, such as lunch breaks, meetings, and helping other anesthesiologists. However, because administering anesthesia is a complicated medical procedure that requires long-term education, extreme caution should be taken as uneducated task shifting may threaten patient safety.

Our hospital has set a 1-year training period for clinical engineers to become CEAAs, after which they have to pass written and oral examinations with a score of at least 70%. Anesthesiologists continue to give lectures and provide on-the-job training even after the program. This education and accreditation system has been applied for > 10 years with many modifications and improvements to meet our environment and needs. Therefore, we recommend beginning task shifting with noninvasive and safe tasks, along with providing education for anesthesia monitoring in the initial phase.

Finally, the limitations of this study must be described. This was a 1-month observational study conducted at a single facility. Therefore, the extent to which the results can be generalized to other facilities is unclear. Moreover, this study was conducted in September, when many conferences were held at the facility; therefore, the results may not be valid throughout the year. Additionally, there were five missing working records. These records were taken from 8:30 a.m. to 5:15 p.m., assuming that there was no overtime duty. However, because we analyzed 228 records, we believe that this does not affect the results. Finally, the total activity time was calculated as the time during which the anesthesiologists were in the hospital. However, this time may include additional break time or on-call time to prepare for a sudden change in a severely ill patient and thus might not be considered labor.

In conclusion, task shift rates > 20% were reported when observing CEAAs at our hospital. Task shifting with CEAAs might be effective in reducing the working hours of anesthesiologists.

## Article Information

### Conflicts of Interest

None

### Author Contributions

YN drafted the manuscript. YN and HK collected data from workers. MK revised the manuscript from the viewpoint of clinical engineers, SO and HI revised the methodology of the research, JE and MK revised the manuscript from the view of anesthesiologists.

All authors contributed and approved the final version of this study.

### Approval by Institutional Review Board (IRB)

This prospective observational study was approved by the Ethics Committee of the Nara Medical University (approval number: 2252)

### 

This study was conducted as part of a study by the Ministry of Health, Labour and Welfare “Research Group on Appropriate Role Sharing of Medical and Care Workers in New Team Medical Care.”

## References

[1] Hoyler M, Finlayson SR, McClain CD, et al. Shortage of doctors, shortage of data: a review of the global surgery, obstetrics, and anesthesia workforce literature. World J Surg. 2014;38(2):269-80. 24218153 10.1007/s00268-013-2324-y

[2] Epiu I, Tindimwebwa JV, Mijumbi C, et al. Challenges of anesthesia in low- and middle-income countries: a cross-sectional survey of access to safe obstetric anesthesia in East Africa. Anesth Analg. 2017;124(1):290-9. 27918334 10.1213/ANE.0000000000001690 PMC5767165

[3] Bharati SJ, Chowdhury T, Gupta N, et al. Anaesthesia in underdeveloped world: Present scenario and future challenges. Niger Med J. 2014;55(1):1-8. 24970961 10.4103/0300-1652.128146 PMC4071655

[4] Matsusaki T, Sakai T. The role of Certified Registered Nurse Anesthetists in the United States. J Anesth. 2011;25(5):734-40. 21717163 10.1007/s00540-011-1193-5

[5] Study Group on Promotion of Task Shifting/Sharing to Promote Work Style Innovation among Doctors. Impact on physicians' work and working hours by nurses who have completed Specified Medical Acts [Internet]. 2019 [cited 2021 Jan 6]. Available from: https://www.mhlw.go.jp/content/10800000/000568281.pdf. Japanese.

[6] Study Group on innovation of the Work Styles of physicians. Report of the Study Group on the innovation of the Work Styles of physicians [Internet]. 2019 [cited 2021 Jan 6]. Available from: https://www.mhlw.go.jp/content/10800000/000496522.pdf. Japanese.

[7] Lim J, Dinges DF. A meta-analysis of the impact of short-term sleep deprivation on cognitive variables. Psychol Bull. 2010;136(3):375-89. 20438143 10.1037/a0018883 PMC3290659

[8] Gaba DM, Howard SK, Jump B. Production pressure in the work environment. California anesthesiologists' attitudes and experiences. Anesthesiology. 1994;81(2):488-500. 8053599 10.1097/00000542-199408000-00028

[9] Gander PH, Merry A, Millar MM, et al. Hours of work and fatigue-related error: a survey of New Zealand anaesthetists. Anaesthesia and intensive care. 2000;28(2):178-83. 10788970 10.1177/0310057X0002800209

[10] Baldwin DC Jr., Daugherty SR. Sleep deprivation and fatigue in residency training: results of a national survey of first- and second-year residents. Sleep. 2004;27(2):217-23. 15124713 10.1093/sleep/27.2.217

[11] Aya AG, Mangin R, Robert C, et al. Increased risk of unintentional dural puncture in night-time obstetric epidural anesthesia. Can J Anaesth. 1999;46(7):665-9. 10442962 10.1007/BF03013955

[12] Kivimaki M, Jokela M, Nyberg ST, et al. Long working hours and risk of coronary heart disease and stroke: a systematic review and meta-analysis of published and unpublished data for 603,838 individuals. Lancet (London, England). 2015;386(10005):1739-46. 10.1016/S0140-6736(15)60295-1 26298822

[13] Meschia JF, Bushnell C, Boden-Albala B, et al. Guidelines for the primary prevention of stroke: a statement for healthcare professionals from the American Heart Association/American Stroke Association. Stroke. 2014;45(12):3754-832. 25355838 10.1161/STR.0000000000000046 PMC5020564

[14] The 3rd Study Group on Promotion of Task Shifting/Sharing to Promote Work Style innovation among physicians. Medical acts available under the current laws [Internet]. 2019 [cited 2021 Jan 6]. Available from: https://www.mhlw.go.jp/content/10800000/000568229.pdf. Japanese.

